# An immunohistochemical study of CD83- and CD1a-positive dendritic cells in the decidua of women with recurrent spontaneous abortion

**DOI:** 10.1186/s40001-014-0076-2

**Published:** 2015-01-07

**Authors:** Zhi-Da Qian, Li-Li Huang, Xiao-Ming Zhu

**Affiliations:** Department of Obstetrics and Gynecology, Women’s Hospital, School of Medicine, Zhejiang University, 1 Xueshi Road, Hangzhou, Zhejiang Province 310006 People’s Republic of China; Key Laboratory of Reproductive Genetics (Zhejiang University), Ministry of Education, Hangzhou, People’s Republic of China

**Keywords:** recurrent spontaneous abortion, decidua, dendritic cells, CD83, CD1a, immunohistochemistry

## Abstract

**Background:**

There are more and more women with recurrent spontaneous abortion (RSA). The mechanism of RSA is still unclear. Immunological factors have been postulated to play a role in the etiology of RSA. Dendritic cells (DCs) are the most potent antigen-presenting cells in the immune system, and the decidual DCs may take part in the occurrence of RSA. The difference in maturity status of decidual DCs among women with RSA and women with normal pregnancies is worthy of studying for its application to prevention and therapy.

**Methods:**

The EnVision two-step immunohistochemical staining technique was used to detect the expression of CD83 and CD1a in the decidua of women with RSA (30 cases) and normal pregnancies (30 cases). The maturity status, distribution and quantity of DCs in the two groups were observed. Observation of the staining and cell counting were done using microscope within 30 randomly selected high-power fields (HPF, 40 × 10). All data analyses were conducted with SPSS 17.0 and the statistical significance was set at *P* <0.05.

**Results:**

The decidua from the two groups contained DCs that stained with the anti-CD83 and anti-CD1a antibody. Most of the decidual CD83^+^DCs from two groups were located in the stroma. There were more CD83^+^DCs clustered with other DCs in the stroma from women with RSA than normal pregnancies. Most of the CD1a^+^DCs in the decidua from the two groups are located close to maternal glandular epithelium. No difference in the location of CD1a^+^DCs was found in the decidua between two groups. The number of decidual CD83^+^DCs was statistically significantly higher in RSA women than in normal early pregnant women (14.20 ± 13.34/30 HPF versus 4.77 ± 2.64/30 HPF; *t* = 3.800, *P* = 0.001). The number of CD1a^+^DCs in the decidua was statistically significantly lower in RSA women compared with normal early pregnant women (3.97 ± 3.75/30 HPF versus 7.60 ± 6.08/30 HPF; *t* = 2.786, *P* = 0.008).

**Conclusions:**

These findings suggest that the increase in the number of mature DCs and the decrease in the quantity of immature DCs in the decidua may be related to RSA. The maturation of decidual DCs may play an important role in the pathogenesis of RSA.

## Background

Miscarriage is the most common complication of pregnancy. Recurrent spontaneous abortion (RSA) is defined as the miscarriage of three or more consecutive pregnancies before 20 weeks of gestation and affects about 1% of infertile couples [1]. Pregnancy is a phenomenon of successful semi-allograft. Its success depends on the mother's immune tolerance of pregnancy. Pathological pregnancy may occur if this tolerance is damaged. The incidence of RSA is influenced by a number of pregnancy-related factors, including anatomic, infectious, endocrine, environmental, genetic and immunologic factors. Immunological factors have been postulated to play a role in the etiology of recurrent miscarriage as the fetus and placenta is semi-allogenic to the mother. Study in the *CBA/J* × *DBA/2 J* mouse model showed that the use of syngeneic DC prevented abortions [2]. There has been a growing interest in the study of immunological factors of RSA. Maternal and fetal immune cells come into direct contact in the decidua, which plays a key role in fetal tolerance. Abnormal immune tolerance of maternal-fetal interface of RSA is related to the dysregulation of human leukocyte antigen (HLA) and apoptosis of natural killer (NK) cells, T lymphocytes, macrophages, dendritic cells (DCs), and other immune cells [3,4].

DCs are the most potent antigen-presenting cells (APC) in the immune system with the unique ability to induce primary immune responses [5]. DCs play an important role in the initiation and regulation of immune responses by regulating T cell-mediated immunity [6,7]. DCs also play an important role in inducing immune tolerance [8]. DCs are derived from bone marrow stem cells, migrate through the blood, and then disperse widely in lymphoid tissues and nonlymphoid tissues, such as liver, heart, kidney and lung tissue (except brain). There are two entities of DCs that differ phenotypically and functionally, the mature and immature DCs (mDCs and iDCs) [7,9]. The iDCs are excellent at antigen uptake, but are poor antigen presenters, and the reverse is true for the mature subgroup. The iDCs transform into mDCs and induce immune response under the influence of mature-signals [7,10]. The differing character in the two groups of DCs is the accessory molecule expression that can be studied immunohistochemically. CD83 is a marker of mDCs [11], and CD1a is a marker of iDCs [12].

DCs exist in normal endometrium and pregnancy decidua [10,13]. Uterine DCs in the decidua have been implicated in pregnancy maintenance. In early pregnancy, PGE2 and IL-10 in the decidua can lead to the generation of tolerant DCs [14,15]. The level of IL-10 in placental tissue gradually increased with the development of normal pregnancy, and high concentrations of IL-10 may inhibit the ability of DCs to produce IL-12, and the balance of the T-helper-1 type response/T-helper-2 type response (Th1/Th2) is shifted to the Th2 direction. The iDCs in the decidua of a normal pregnancy do not express CD83 molecules [16]. This suggests that the formation of maternal-fetal immune tolerance may be related to the immature status of DCs in the microenvironment of the maternal-fetal interface. Blois *et al*. [2] studied the effect of adoptive transfer of DCs on the maintenance of pregnancy in the *CBA/J* × *DBA/2 J* mouse model. They found that the control (no treatment) abortion rate was 23.8%, and with GM-CSF alone was 17.6%. The abortion rate was reduced to 2.2% after inoculation of syngeneic DCs. It suggested that syngeneic DCs may have a significant protective effect in miscarriage in pregnant mice. DCs may not only have mediated the defensive immune response, but also the tolerance of embryos. There was a fine balance in the interaction between DCs and trophoblast cells during successful pregnancy in mice. DCs therapy could upregulate a regulatory/protective population of cells at the maternal-fetal interface [17,18]. Askelund *et al*. [19] found there were significantly more CD83^+^DCs in the decidua from women with RSA than normal pregnancies at 8 weeks’ gestation. It suggested that DCs may be associated with any immune-related pathological pregnancy, and DCs may play an important role in the pathogenesis of RSA. However, little is known about the underlying mechanisms. The difference in maturity status of decidual DCs between women with RSA and normal pregnancies is worthy of studying for its application to prevention and therapy. This study examines the mature and immature DC density using CD83 and CD1a antibody, respectively.

## Methods

### Patients

This is a prospective study of DCs in the uterine decidua. The specimens were obtained from the Department of Obstetrics and Gynecology, Women’s Hospital, School of Medicine, Zhejiang University, China. Samples of RSA group (30 cases) were collected from women with RSA by ultrasound guided curettage immediately upon confirmation of embryonic demise. Time delay between embryonic demise and curettage was no more than 2 days. Samples of the control group (30 cases) were collected from women who underwent elective termination of normal pregnancies without history of miscarriages in the same hospital. All women were from 20 to 40 years of age with gestation of less than 14 weeks. There was no significant difference between the general information of the two groups (Table 1). Excluded from the study were women with genetic, endocrine and immune disorders, cancer, genital anatomical abnormalities and major surgical diseases, history of long-term medication, trophoblastic disease and those undergoing endocrine therapy, chemotherapy, and radiotherapy. The products of conception and the parents were karyotyped to exclude chromosomal abnormalities in the RSA group. Written informed consent was obtained from each patient and the study was approved by the Institutional Review Board of Women’s Hospital, School of Medicine, Zhejiang University, China.

**Table 1:** **General information for the two groups**

	**N**	**Age (years; mean ± SD)**	**Gestational age (days; mean ± SD)**	**Abortions (mean ± SD)**
RSA	30	29.77 ± 4.45	64.17 ± 11.36	4.33 ± 1.52
Normal pregnancy	30	28.87 ± 5.84	59.03 ± 9.35	3.93 ± 0.52
*P*		0.504	0.073	0.180

There was no significant difference between the general information for the two groups. RSA, recurrent spontaneous abortion.

### Immunohistochemistry

Decidua were immediately placed into a 10% formaldehyde solution bag and fixed for 10 to 24 hours. Paraffin blocks were sectioned into a 4-μm thickness. The EnVision two-step immunohistochemical staining technique was used to detect the expression of CD83 and CD1a in the decidua. The immunohistochemical staining was done by one person only. Positive controls were included in every batch of tests. Primary antibodies: Mouse antihuman CD83 (Model NCL-CD83, clone number 1H4b) was obtained from Novocastra Biotechnology Co., Ltd, US. Mouse antihuman CD1a (Model GM357104, clone number 010) was obtained from DAKO Biotechnology Co., Ltd, Denmark. The secondary antibody used was ChemMatei Envisioni Detection Kit Peroxidase/DAB, from DAKO Biotechnology Co., Ltd, Denmark. The normal tonsil was used as a CD83 positive control, and the normal skin was used as a CD1a positive control.

### Dendritic cell counts

DCs displayed membrane staining, cytoplasmic staining, and branch-like morphological features. MDCs were characterized by the expression of CD83, and iDCs were characterized by the expression of CD1a. The location and number of DCs in the two groups were observed within 30 randomly selected high-power fields (HPF, ×40 objective and × 10 eye piece). Observation of the staining and cell counting were done using a Olympus CHK microscope (Olympus, Japan). Photomicrographs were taken using a digital camera (Nikon, Japan). All the immunostained slides were reviewed by two observers independently.

### Statistics

The difference in the counts of DCs between the two groups was evaluated. All data analyses were conducted with SPSS 17.0 (SPSS Inc., USA). Statistical significance was set at *P* <0.05, and *P* values from all tests were reported. The statistical significance of the experimental differences in the two groups was assessed by a normal distribution test first. If the data were normally distributed, an independent samples *t* test for continuous variables analysis was used, and if the data were not normally distributed, a non-parametric test (Mann-Whitney Test) was used.

## Results

### The location of decidual dendritic cells

**Figure 1:**
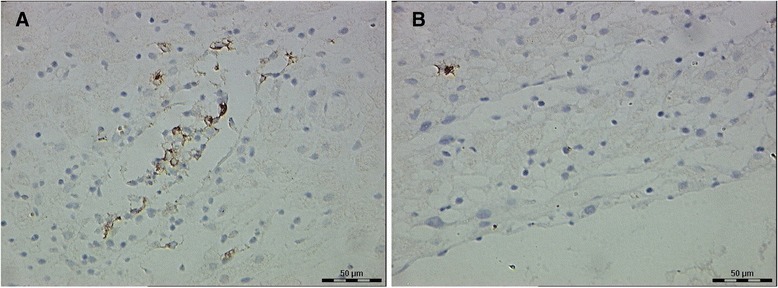
**CD83** ^**+**^ **dendritic cells (DCs) in the decidua of the two groups. (A)** Decidua of recurrent spontaneous abortion (RSA) (a few of CD83^+^DCs clustered in the stroma, 40 × 10). **(B)** Decidua of normal pregnancies (a single CD83^+^DC in the stroma, with membrane staining, cytoplasmic staining and branch-like morphology, 40 × 10).

**Figure 2:**
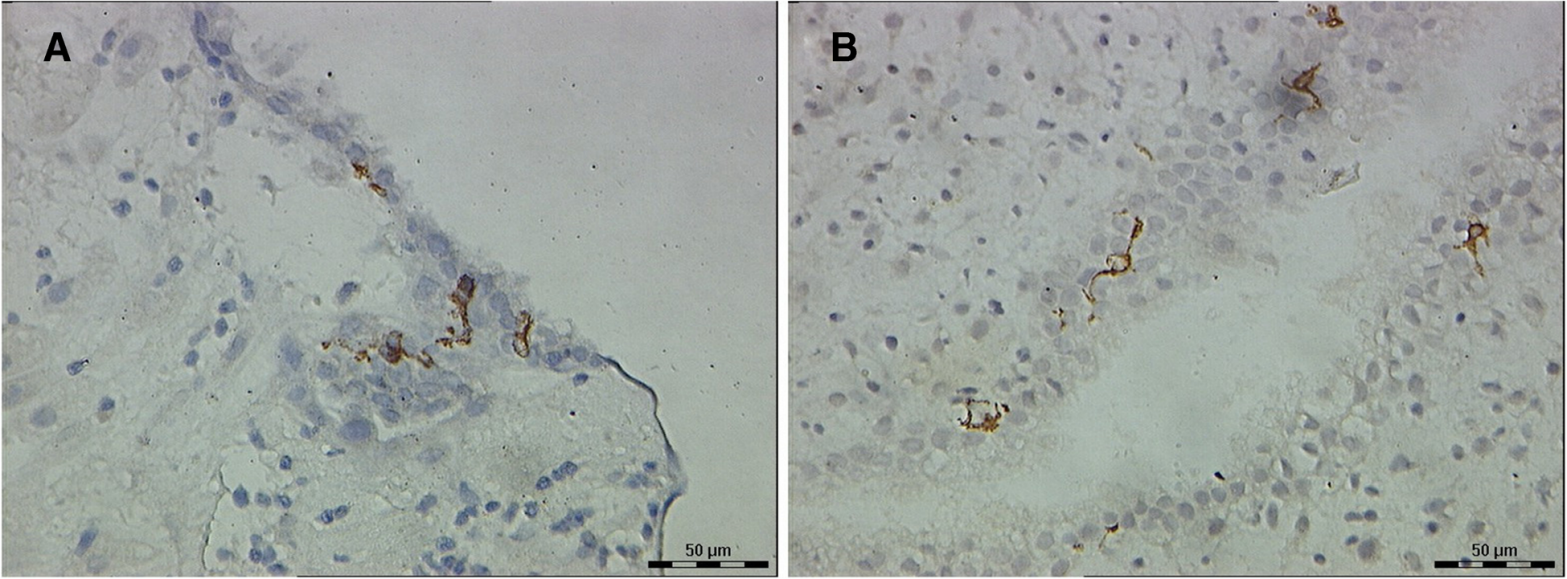
**CD1a** ^**+**^ **dendritic cells (DCs) in the decidua of the two groups. (A)** Decidua of recurrent spontaneous abortion (RSA) (some CD1a^+^DCs were located close to uterine epithelium, 40 × 10). **(B)** Decidua of normal pregnancies (a few of CD1a^+^DCs were located close to glandular epithelium, with membrane staining, cytoplasmic staining and branch-like morphology, 40 × 10).

CD83^+^ DCs were found in decidual samples from both RSA and normal pregnancies. DCs displayed membrane staining, cytoplasmic staining, and appropriate morphological features. Most of the CD83^+^DCs in the decidua from women with RSA were clustered with other DCs (Figure 1A), and few of them were present as single cells in the stroma. The localization of CD83^+^DCs in the decidua of women with normal pregnancies was similar to that seen for women with RSA. However, most of the decidual CD83^+^DCs from women with normal pregnancies were present as single cells in the stroma and were not clustered with other DCs (Figure 1B). Very few of CD83^+^DCs were located close to maternal glandular epithelium. The decidua from all groups were found to contain DCs that stained with the anti-CD1a antibody. CD1a^+^ DCs displayed membrane staining, cytoplasmic staining, and branch-like morphological features. Most of the decidual CD1a^+^DCs in the two groups were located close to maternal glandular epithelium and uterine epithelium (Figure 2A, B). There was no difference in the location of CD1a^+^ DCs in the decidua between the two groups.

### Dendritic cell counts

The number of CD83^+^DCs of decidual tissue from women with RSA was 14.20 ± 13.34/30 HPF and from women with normal pregnancies was 4.77 ± 2.64/30 HPF. There were significantly more CD83^+^DCs in the decidua of women with RSA than in women with normal pregnancies (*t* = 3.800, *P* = 0.001). The number of decidual CD1a^+^ DCs in RSA and normal pregnancies groups were 3.97 ± 3.75/30HPF and 7.60 ± 6.08/30HPF, respectively. There were more CD1a^+^DCs in the decidua from normal pregnancies than RSA, and the difference was significant (*t* = 2.786, *P* = 0.008) .

## Discussion

The maternal-fetal relationship is a unique immunologic phenomenon. Our research revealed that most of the mDCs in the decidua from the two groups were located in the stroma. There were more CD83^+^DCs clustered with other DCs in the stroma from women with RSA than from women with normal pregnancies. CD83^+^DCs’ aggregate suggests that these DCs may be immunologically active. Our results were somewhat different from the results of others. Askelund *et al*. [19] reported that most of the CD83^+^DCs in the decidua from women with RSA and normal pregnancies were present as single cells in the stroma. It might be that many of the DCs distributed randomly throughout the decidual stroma were also in close proximity to T cells so they might also be immunologically active. The difference may be due to the different maturation status of DCs and the small number of decidual samples in both studies. Kammerer *et al*. [20] found that CD83^+^ cells were localized to the basal layer of the decidua in normal early pregnancies. It may be due to our decidual samplings, which were more superficial. It is also possible that the different distribution of DCs throughout the decidua may represent the early stages of a maternal immune response to the failed pregnancy, rather than a cause of pregnancy failure. However, this seems unlikely as the time delay between embryonic demise and curettage was no more than 2 days in our study.

CD1a^+^ DCs were found in both decidual samples of RSA and normal pregnancies. Most of the decidual CD1a^+^DCs in two groups were located close to maternal glandular epithelium or uterine epithelium and there was no difference between two groups. There were some discrepancies between our data and those from Gardner *et al*. [13], who reported an absence of CD1a expression in decidual leukocytes and no cells in the stroma stained for CD1a at 7 to 12 weeks gestation. The difference between our results and those of Gardner *et al*. may be due to the different experimental method (immunofluorescence labeling for flow cytometry) used by Gardner *et al*. We consider that the glandular epithelium and uterine epithelium are the primary position of the local maternal-fetal immune response. Maternal glandular epithelium is close to gestational sac (the maternal-fetal interface). DCs and other immune cells may be involved in gathering the local immune response in the uterine decidua during the embryo’s implantation.

This investigation showed that the mean decidual CD83^+^DCs population in RSA was higher than the mean population in the normal pregnancies and was statistically significant. This observation suggests the increase in the number of mDCs probably related to the pathogenesis of RSA. This is different to the findings by Askelund *et al*. [19]. They did not find significant differences in DCs numbers between the women with recurrent miscarriage and women with normal pregnancies. We assume that an active immune response exists in the RSA decidua. The maturation of DCs determines the outcome of immune response. IDCs can lead to immune tolerance in the physiological condition and require a maturation step that promotes antigen presentation to prime the T cell activity and trigger the immune response. IDCs transform into mDCs and induce immune response under the influence of mature-signals (such as bacterial DNA, proinflammatory cytokines, etc.). DCs initiate the function of T cell strongly at this time.

DCs are responsible for stimulating virgin T cells, a property that distinguishes them from other APC. The action of cytotoxic T cells in large part determines whether an allograft survives or is rejected. DCs are involved in regulating the balance between Th1 and Th2, and the Th1/Th2 balance regulates the maintenance of the pregnancy. The maternal balance between Th1 and Th2 type cytokines contributes to the success of the pregnancy [21,22]. Th1-type cytokines (such as IL-2, IL-12, IFN-γ, TNF-β, etc.) can defend against microbial infections, and also may lead to abortion. For example, IL-12 can promote a T cell to Th1-type biased, induced immune response. However, Th2-type cytokines (such as IL-4, IL-5, IL-10, etc.) can lead to less autoimmunity, susceptible to infections, and also may induce pregnancy-tolerance to fetus, providing protection to pregnancy. For instance, IL-10 can induce T-cell regulation (regulatory T cells, Treg) and promote monocyte-derived DCs induce antigen-specific CD4^+^ and CD8^+^ T cell immune incompetence [23], which can inhibit immune response of T-cell and induce tolerance of T cell [24].

Research on mouse models [25] showed that fetal antigens on the placenta during the implant period could stimulate DCs in the decidua to produce Th1-type cytokines. Th2-type cytokines then dominated. The level of IL-10 in placenta gradually increased with the development of pregnancy in normal pregnancy. High concentrations of IL-10 may inhibit the decidual DCs' ability to produce IL-12 and then regulates the Th1/Th2 balance to Th2 and shift the direction of the end-mediated immune tolerance to the fetus. Decidual iDCs uptake antigens and then transform into mDCs with antigen presenting function, which can induce and stimulate the proliferation of naive T cells. The maternal immune response breaks the balance between Th1/Th2, and makes the maternal immune response to Th1-type. The above reaction leads to a semi-allograft immune response to the fetal and placenta by mother and occurrence of miscarriage eventually.

IDCs are characterized by the CD1a expression. We found lesser CD1a^+^DCs in the decidua of RSA than in the decidua from normal pregnancies. This suggests that the decrease in the quantity of iDCs is probably related to the occurrence of RSA. It is possible that decidual iDCs play a role in the maintenance of normal pregnancy through the regulation of maternal-fetal immune tolerance [26]. Most of the DCs in the nonlymphoid organs are immature. Studies found that some DCs subtypes in human decidua may play an important role to maintain pregnancy [27,28]. Decidual DCs may have a significant protective effect in miscarriage in pregnant mice [2]. Therapy with DCs can reduce the spontaneous abortion rate, through a mechanism whereby DCs therapy differentially upregulated a regulatory/protective population of cells at the fetal-maternal interface [18].

DCs may regulate the Treg cells to suppress the semi-allograft rejection from mother to the fetus and play an important role in the maternal-fetal immune tolerance. Treg cells have significant roles in the negative regulation of immune response and induction of immune tolerance. CD4^+^CD25^+^ Treg cells exist in both maternal blood and decidua throughout pregnancy. Zenclussen *et al*. [29] reported Treg cells were proposed to play an essential role. Normal pregnant mice show an expansion of CD4^+^CD25^+^ and IL-10^+^ Treg cells at the periphery compared to nonpregnant animals. Further, they suggested significantly lower frequencies of Treg cells in abortion-prone mice. CD4^+^CD25^+^ Treg cells from normal pregnant mice were able to prevent fetal rejection. Accordingly, downregulated levels of Treg cells were also reported during human miscarriage. There was an increase in circulating Treg cells during early pregnancy, peaking during the second-trimester [30]. In peripheral blood, statistically significantly higher proportions of CD4^+^CD25^+^T cells expression was observed in normal early pregnant women compared with normal nonpregnant women and unexplained RSA patients. There were statistically significantly lower proportions of CD4^+^CD25^+^ T cells expression in unexplained RSA patients compared with normal early pregnant women in their decidua [31].

IDCs inhibit proliferation of antigen-specific T cell by self-secretion of IL-10. IDCs induce CD4^+^CD25^+^ Treg and CD8^+^ suppressor T cells, which inhibit the immune response [32]. IL-10 secreted by DCs was increased when there is interaction between DCs and Treg cells. DCs and Treg cells formed a feedback inhibition [33]. IDCs in the peripheral tissue induce removal of antigen-specific T cells and induce peripheral tolerance under normal physiological conditions without infection and tissue damage. The immune system identifies and defends ‘danger signals’ from tissue damage to maintain tolerance of self-antigens and harmless exogenous antigens through this mechanism.

## Conclusions

In spite of an increasing interest, the etiology of RSA remains unknown in approximately 50 percent of cases. This study demonstrates significant associations between the maturation of decidual DCs and RSA. The increase in the number of mDCs and the decrease in the number of iDCs may be related to RSA. As mDCs are important antigen presenters, these observations suggest the existence of an active immune response in the decidua of women with RSA. DCs play an important role in the aetiology of RSA. However, its specific pathogenesis has not been clarified exactly yet, and the immunologic nature of RSA has generated considerable interest and controversy. Many factors could be correlated to RSA. For example, passive smoking, a higher body mass index (BMI), and a family history of RSA were thought to be independent risk factors for RSA [34]. Further researches are needed to characterize the specific functions of decidual DCs. Despite awareness of the limitations of this study due to the small sample size and semi-quantitative evaluation, the results encourage further larger studies in this promising research field.

## Consent

Written informed consent was obtained from each of the patients for publication of this report and any accompanying images. A copy of this written consent is available from each patient for review by the Editor-in-Chief of this journal.

## Abbreviations

APC: antigen-presenting cells
BMI: body mass index
DCs: dendritic cells
HLA: human leukocyte antigen
HPF: high-power fields
iDCs: immature DCs
mDCs: mature DCs
NK cells: natural killer cells
RSA: recurrent spontaneous abortion
Th1: T-helper-1 type response
Th2: T-helper-2 type response
Treg: regulatory T cells
